# Anti-herpes virus activity of the carnivorous botanical, *Sarracenia purpurea*

**DOI:** 10.1038/s41598-020-76151-w

**Published:** 2020-11-03

**Authors:** Latha Kannan, Ashok Kumar, Aradhana Kumar, Bertram Jacobs, Jeffrey Langland

**Affiliations:** 1 Ric Scalzo Institute for Botanical Research, Southwest College of Naturopathic Medicine and Health Sciences, Tempe, AZ 85282 USA; 2 Biodesign Institute, Arizona State University, Tempe, AZ 85287 USA; 3 College of Health Solutions, Arizona State University, Phoenix, AZ 85004 USA

**Keywords:** Antimicrobials, Virology

## Abstract

Herpes simplex virus type-1 (HSV-1), one of the most widely spread human viruses in the Herpesviridae family, causes herpes labialis (cold sores) and keratitis (inflammation of the cornea). Conventional treatment for HSV-1 infection includes pharmaceutical drugs, such as acyclovir and docosonal, which are efficacious but maintain the potential for the development of viral drug resistance. Extracts from the carnivorous pitcher plant, *Sarracenia purpurea,* have previously been shown to inhibit the replication of HSV-1. In this study, we demonstrate that *S. purpurea* extracts can inhibit the replication of HSV-1 by two distinct mechanisms of action. These extracts directly inhibit extracellular virions or viral attachment to the human host cell as well as inhibiting the expression of viral immediate-early, early and late genes when added at various times post-infection. This botanical has previously been shown to inhibit the replication of poxviruses through the inhibition of early viral gene transcription. These results support a broader anti-viral activity of *S. purpurea* extracts against both pox and herpes viruses.

## Introduction

According to the World Health Organization, 90% of the human population is infected with Herpesvirus family members, including herpes simplex virus type-1 (HSV-1). HSV-1 is a highly infectious virus that causes the primary infection, herpes labialis, and establishes a latent infection in the neural ganglia^1,2^. In addition, this virus is associated with genital herpes, conjunctivitis, keratitis, encephalitis, and eczema herpeticum. The virus is highly prevalent and endemic worldwide. Sixty-seven percentage of global population of ages 0–49 years are infected with HSV-1, with highest prevalence in Africa, South-East Asia and the western Pacific^3–5^. During initial infection, the viral DNA is transported to the spinal cord sensory ganglion through axon transport and latency is established. While in latency, the viral lytic genes are suppressed, and the genome is maintained as a small circular extra chromosomal episome. Occasionally, the viral genome in the ganglia reactivate and the virus migrates back through axons to the original site of infection^6,7^. HSV-1 is also associated with more severe infections in neonates, elderly people, patients with acquired immune deficiency syndrome, and patients with drug-induced immune suppression.

Current available treatments for HSV-1 include acyclovir and its derivatives, such as famciclovir and valacyclovir. Acyclovir, a guanine nucleoside analog, competitively targets the viral DNA polymerase, resulting in chain termination and preventing viral DNA elongation^8^. The effectiveness of these drugs, however, are limited in immune-suppressed patients, resulting in increased likelihood of the virus to develop drug resistance^9,10^. In addition, these drugs also exhibit side effects including nausea, diarrhea, and vomiting. Drugs like foscarnet, a pyrophosphate analog, and cidofovir, a nucleotide analog, can be used when acyclovir-resistance has developed, although these drugs display reduced bioavailability and nephrotoxicity, respectively^11–14^. HSV-1 develops drug resistance in patients predominantly due to mutations in the genetic code for thymidine kinase as well as DNA polymerase^15^. Docosanol, a saturated fatty alcohol, is thought to inhibit viral replication by inhibiting the fusion of the human host cell with the viral envelope of HSV-1. Clinical trials demonstrated that this drug reduced the healing time of herpes labialis lesions by only 17.5 h on average. Based on these available therapies, developing novel treatments against HSV-1 infection would be highly beneficial. Medicinal plants contain an abundance of natural compounds and have been used traditionally throughout history in many countries to treat viral infections^16–21^. Plants such as *Sarracenia purpurea* (*S*. *purpurea*), *Melissa officinalis*, *Clinacanthus nutans, Glycyrrhiza glabra*, *Rhus chinensis, Rhus javanica*, and *Punica granatum* have been reported to contain anti-herpetic activity^22–33^. These medicinal plants may possess potential anti-herpetic compounds to treat recurrent HSV-1 infection. The current study investigated the anti-herpetic activity of S. *purpurea* in HSV-1 infected Vero cells. *S*. *purpurea* (commonly known as purple pitcher plant) is a carnivorous plant mainly found on the Eastern seaboard and Gulf Coast of the United States and most of Canada. This plant has been used in traditional medicine for a wide variety of medical illnesses, including smallpox infection, gynecological problems, diabetic problems, mycobacterial infection, and liver/kidney complaints^34–37^. Our lab has previously demonstrated that extracts from *S. purpurea* have the ability to inhibit the replication of poxviruses by inhibiting early viral transcription^34^. This study also demonstrated that *S. purpurea* extracts have broad antiviral activity and inhibit the replication of HSV-1. Limited clinical trials for HSV-1 infection, performed by three different research groups, determined that a topical application of *S. purpurea*, provided rapid relief from the pain and improved healing of the viral-associated lesions, as compared to the placebo group^38–40^. These results support that S. *purpurea* may have bioactive anti-herpes components which may effectively treat recurrent HSV-1 symptoms.

Infection by HSV-1 is facilitated through viral surface glycoproteins, gC, gB, gD, gH and gL, which are present in the viral envelope. Initial host cell binding occurs via gC and gB which bind to cell surface glycosaminoglycans, heparan sulfate, and chondroitin sulfate, or through interaction between gC and the scavenger receptor, MARCO^41–44^. High affinity gD then binds to the receptors, nectin-1, nectin-2, HVEM, or 3-O-sulphated heparan sulfate, inducing a conformational change and initiating membrane fusion through interaction with the gB and gH/gL complex^45–49^. Together, this glycoprotein complex as well as cell surface receptors mediate membrane fusion and the release of viral particles into the host cell^50,51^. During HSV-1 replication, viral gene expression is complex and occurs sequentially in stages identified as immediate-early, early, and late genes. The immediate-early genes are typically involved in controlling host cell function, for example, ICP4 plays a significant role in the inhibition of host gene transcription. The viral early proteins are generally involved in DNA replication where, for example, ICP8 stimulates viral DNA replication^52,53^. The late proteins form the capsid and the receptors on the surface of the virus. As mentioned, the glycoprotein, gC, plays a vital role in adsorption of the virus to the host cell.

In the current study, we demonstrate that *S. purpurea* extracts can inhibit the replication of HSV-1 through two distinct mechanisms of action. The extracts directly inhibit extracellular virions or viral attachment to the host cell as well as inhibiting the expression of ICP4, ICP8 and gC when added at various times post-infection. These results support the broader anti-viral activity of *S. purpurea* extracts against both pox and herpes viruses.

## Materials and methods

### Cell line and virus stocks

Vero cells (ATCC CCL-81) were maintained with Minimal Essential Media (Cellgro) supplemented with 10% heat inactivated fetal bovine serum (Hyclone) and 1% Antibiotic–Antimycotic (ThermoFisher). Cells were incubated at 37 °C, with 5% CO_2_ in a humidified chamber. HSV-1 KOS (a kind gift from David Bloom, Univ. of Florida College of Medicine) was propagated in Vero cells.

### Botanical extracts

For the preparation of *S. purpurea* extract, fresh whole plants, grown in a greenhouse in the Southeastern United States, were shipped overnight express. All plant material was subsequently verified by qualified botanical specialists using reference keys. A voucher specimen of all plant material was deposited in a repository. The plants were manually cleaned on the same day received, with special attention to cleaning the base portion of the plant’s pitcher structure so that it was free from contamination with forest detritus and insects. The entire aerial portion/pitcher of the plant was dried (at room temperature for 5 days) and then ground to a fine powder in a VitaMix blender. The plant material was extracted overnight at room temperature with constant mixing in 50% ethanol, 10% glycerin (1:15 weight:volume). The plant/liquid mixture was centrifuged at 3000 × *g* for 15 min and the supernatant filtered through a 0.2 µm syringe filter. The final extract was stored at room temperature in a sterile container.

### Plaque reduction assay

Vero cells were infected with 100–200 (plaque forming units) pfu of HSV-1 KOS in the presence of increasing concentrations of *S. purpurea* or vehicle (50% ethanol, 10% glycerin) for 1 h at 37 °C followed by incubation in media containing *S. purpurea* or vehicle (50% ethanol, 10% glycerin) for 3 days at 37 °C. Plaques were visualized by staining with 0.1% crystal violet in 20% ethanol. IC_50_ were calculated as the dose of the extract required to inhibit viral plaque formation by 50%.

### Cell viability assay

Vero cells were treated with *S. purpurea* or vehicle (50% ethanol, 10% glycerin) at the concentrations indicated. Viability was determined using an MTS assay (abcam) at 24 h post treatment. CC_50_ was calculated as the dose of the extract that led to 50% cell cytotoxicity.

### Single cycle growth curves

Vero cells were infected with HSV-1 KOS at a multiplicity of infection (MOI) of 5 with increasing concentrations of *S. purpurea* or vehicle for 1 h at 37 °C. Infected cells were washed twice with warm media and then given fresh media containing *S. purpurea*. Infected cells were harvested at 1 h (input virus) and 24 h post infection (h.p.i.) by scraping into the media. Infected cells were pelleted at 3000 × *g* for 10 min, suspended in 10 mM Tris, pH 9.0 and stored at − 80 °C. Samples were freeze-thawed three times and titered by plaque assay.

### Western blots

Vero cells were infected with HSV-1 KOS at a MOI of 5. *S. purpurea* was added to the cells at various times following infection (0, 1, 2, 4, 6 h.p.i.). At 16 h.p.i., cells were washed two times with PBS and lysed with 1X SDS sample buffer [125 mM Tris–Cl, pH 6.8, 25% glycerol, 2.5% SDS, 100 mM β-mercaptoethanol, 0.025% bromophenol blue, 10% protease inhibitor cocktail (ThermoScientific)] and heated for 10 min at 95 °C. Samples were separated on 10% polyacrylamide gels, transferred to nitrocellulose membrane in blotting transfer buffer (10 mM CAPS buffer pH 11.0, 20% methanol) and blocked with 25 mM Tris, pH 7.5, 137 mM NaCl, 2.5 mM KCl, 0.025% Tween, 5% powdered milk. Antibodies for ICP4, ICP8, and gC (Abcam) and actin (Santa Cruz) were diluted as per manufacturer’s specifications. Detection was performed using goat anti-mouse or anti-rabbit IgG secondary conjugated to horseradish peroxidase (Santa Cruz) in the presence of a chemiluminescent substrate (ThermoFisher). Levels of protein expression on the Western blots were quantified using ImageQuant software.

### Cell binding/attachment assay

*Sarracenia purpurea* effects on HSV-1 binding/attachment to Vero cells was assayed by different protocols. HSV-1 cellular attachment was measured by adding 200 pfu HSV-1 KOS with increasing concentrations of *S. purpurea* and infecting pre-chilled Vero cell monolayers followed by incubation for 2 h at 4 °C to allow binding (but not cellular uptake). Following incubation, cells were washed two times with cold PBS to remove unbound virus, followed by the addition of complete media. The cells incubated for 3 days at 37 °C and crystal violet staining to visualize plaque formation.

Free-virus treatment was performed using 200 pfu of HSV-1 KOS treated with increasing concentrations of *S. purpurea* and incubation for 1 h at room temperature. Following incubation, the virus was pelleted by centrifugation at 20,000 × *g* for 1 h, washed with media, and resuspended in complete media. Vero cells were infected with the viral sample for 1 h, washed twice with media to remove unbound virus, and fresh media added to cells and incubated for 3 days at 37 °C to observe plaque formation.

Cell pre-treatment was performed by treating Vero cell monolayers with increasing concentrations of *S. purpurea* for 1 h at 37 °C. The monolayers were then washed three times with media to remove unbound extract. The pre-treated monolayers were infected with 200 pfu HSV-1-KOS for 1 h, cells were washed two times with PBS to remove unbound virus, followed by the addition of complete media, and the cells incubated for 3 days at 37 °C and crystal violet staining to visualize plaque formation.

### Real-time PCR

Vero cells were infected with HSV-1 KOS at a MOI of 5. *S. purpurea* was added at various times post infection (0, 1, 2, 4, 6 h.p.i.). At 8 h.p.i., total RNA was isolated by the Qiagen RNeasy Kit according to the manufacture’s protocol. Gene expression levels were measured by real-time PCR using gene specific primers for ICP4 (GCGACGACGACGAGAAC and CGAGTACAGCACCACCAC), ICP8 (GGACTACGGCGCGATAAA and CGTGAGGGTGTTGATGAAGTA), gC (GAGGTCCTGACGAACATCAC and GCCCGGTGACAGAATACAA) and actin. Gene expression levels were normalized to actin.

## Results

### Extract of *S. purpurea* inhibited HSV-1 replication

**Figure 1:**
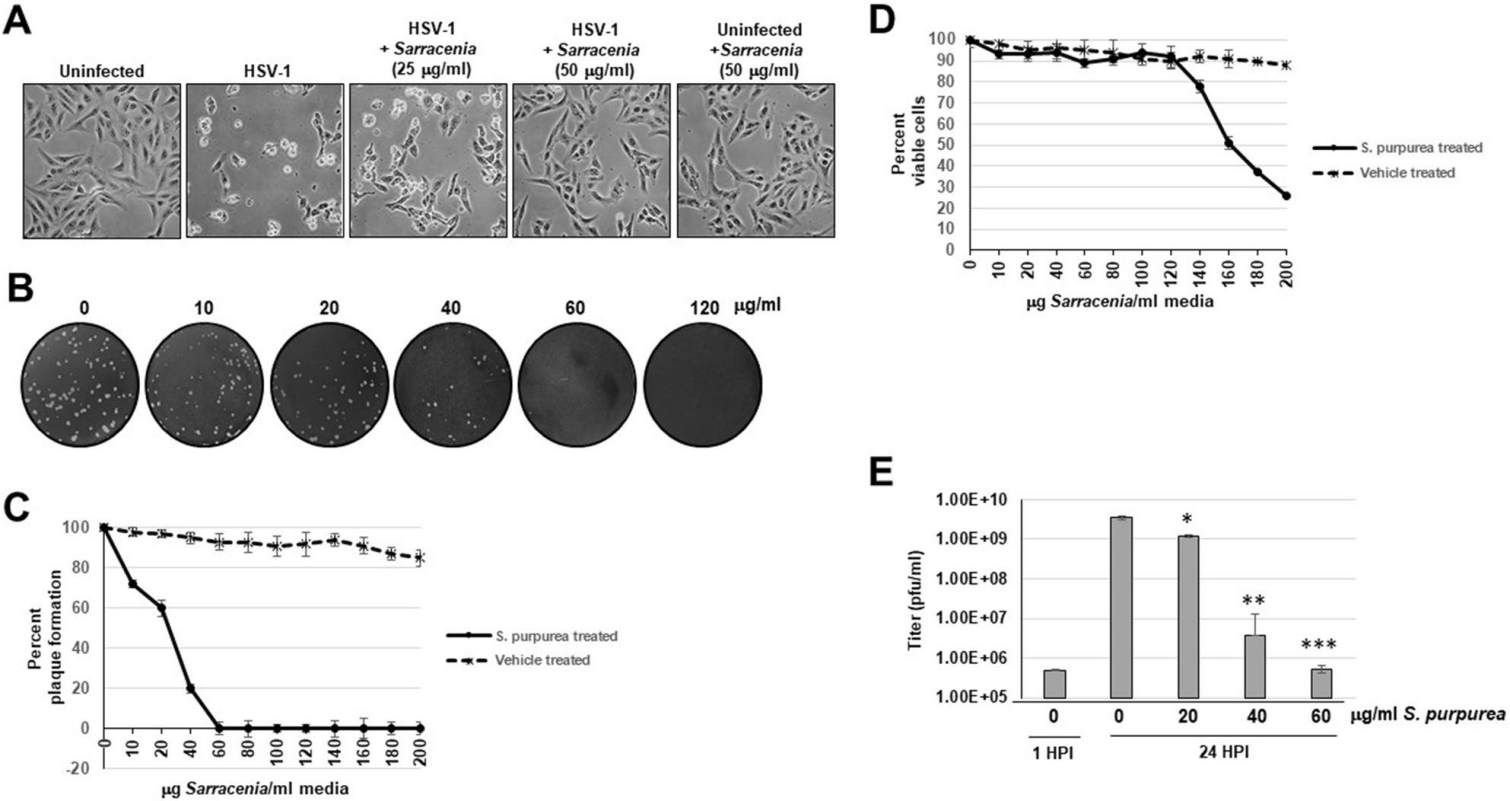
*S. purpurea* inhibited HSV-1 induced cytopathic effect and replication. (**A**) Vero cells were mock infected or infected with HSV-1 at a MOI = 5 and treated with 25 and 50 µg/ml of *S. purpurea* extract. The cell monolayers were photographed at 24 h.p.i. (**B**) Vero cells were infected with 100 pfu HSV-1 and treated with 0, 10, 20, 40, 60, or 120 µg/ml *S. purpurea* extract for 3 days. After 3 days, the cell monolayers were stained with crystal violet. (**C**) The plaque assay in (**B**) was repeated in the presence of the *S. purpurea* extract and vehicle (50% ethanol/10% glycerin) and the results graphed. Error bars indicate the standard deviation from three separate trials. (**D**) Vero cells were treated with increasing concentrations of *S. purpurea* extract or vehicle and cell viability measured at 24 h post-treatment and the results graphed. Error bars indicate the standard deviation from three separate trials. (**E**) Vero cells were infected with HSV-1 at a MOI = 5 and treated with 0, 20, 40, or 60 µg/ml of *S. purpurea* extract. Virus were harvested at 1 (for input virus titer) and 24 h.p.i. and titered. Statistical analysis was performed using a paired t-test. Samples with statistically significant deviation relative to the 0 μg/ml *S. purpurea* treatment are indicated with asterisks (*p < 0.05, **p < 0.01, ***p < 0.005).

Our previous studies demonstrated that *S. purpurea* extracts could inhibit the accumulation of HSV-1 proteins suggesting an inhibition of viral replication^33^. To confirm and further characterize that *S. purpurea* extracts could inhibit HSV-1 replication, Vero cells infected with HSV-1 or uninfected were treated in the presence or absence of *S. purpurea* extracts and monitored for cytopathic effect (CPE). As shown in Fig. 1A, HSV-1 infection induced observable CPE after 24 h. When virally infected cells were treated with increasing doses of the extract, this CPE was moderately to fully inhibited (Fig. 1A). In addition, treatment with *S. purpurea* extract alone did not induce any noticeable cell toxicity (Fig. 1A). This agrees with our previous studies on the effects of *S. purpurea* on poxviruses^34^. To quantitate this anti-HSV-1 effect, a plaque reduction assay was performed. As shown in Fig. 1B, a dose dependent reduction in plaque formation was observed with a 50% reduction in plaques observable at approximately 30 μg/ml. For clarity, this concentration of the *S. purpurea* extract (in μg/ml) represents the concentration of total non-volatile components present in the total plant extract and not the concentration of the active compound since this is unknown at this time. The IC_50_ for the *S. purpurea* extract based on plaque reduction was calculated to be approximately 23 μg/ml and the CC_50_ using an MTS assay was calculated to be approximately 161 μg/ml resulting in a Selectivity Index of 7 (Fig. 1C,D). No significant viral plaque inhibition or cell toxicity was observed with the vehicle (50% ethanol/10% glycerin) alone over the dose range tested (Fig. 1C,D). To further confirm the anti-HSV-1 activity of *S. purpurea*, a single-step growth curve experiment was performed. Vero cell were infected with HSV-1 at a MOI of 5 and treated with increasing concentrations of *S. purpurea* extract. After 24 h, the viral yield was determined. Untreated HSV-1 infection gave an approximate 4-log increase in viral titer compared to the input virus (1 h.p.i.) (Fig. 1E). Treatment with *S. purpurea* gave a dose-dependent reduction in viral titers with an approximate 3-log reduction at 40 μg/ml and a 4-log reduction at 60 μg/ml. (Fig. 1E). These results together confirm the anti-HSV-1 activity of *S. purpurea* extracts.

### Temporal effects of *S. purpurea* extract on the inhibition of HSV-1 replication

**Figure 2:**
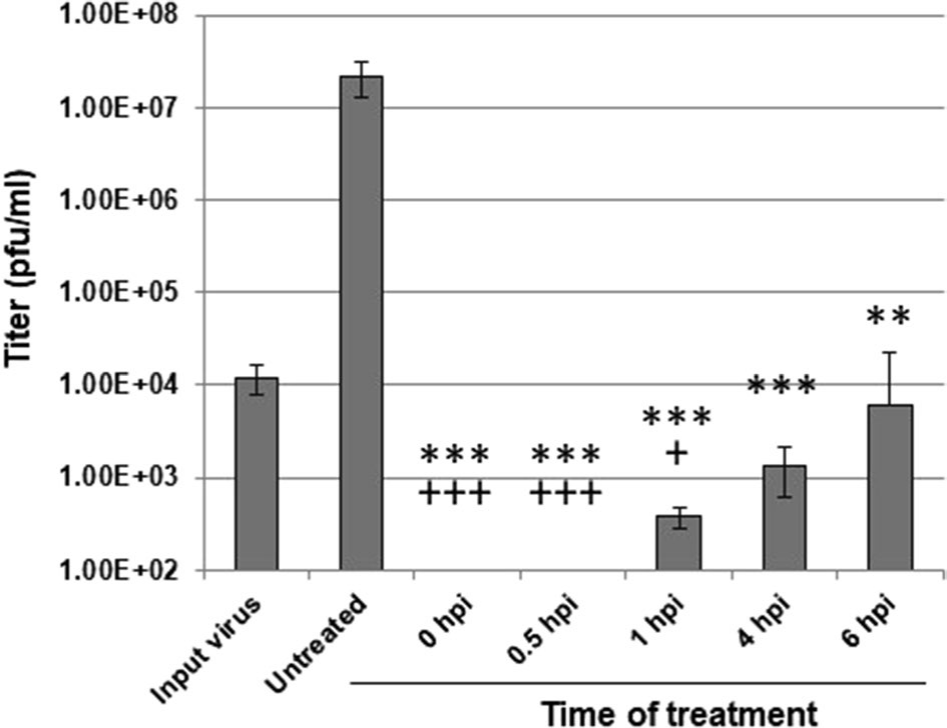
*S. purpurea* temporal inhibition of HSV-1 replication. Vero cells were infected with HSV-1 at a MOI = 5 in the presence or absence of *S. purpurea* (40 µg/ml) added at the indicated times post-infection. At 24 h.p.i, virus was harvested and titered. Input virus was harvested at 1 h.p.i. Error bars indicate the standard deviation from three separate trials. Statistical analysis was performed using a paired t-test. Samples with statistically significant deviation relative to the Untreated sample are indicated with the asterisk (*p < 0.05, **p < 0.01, ***p < 0.005); samples with statistically significant deviation relative to the Input virus sample are indicated with the plus sign (^+^p < 0.05, ^+^p < 0.01, ^+^p < 0.005).

To begin deciphering the mechanism of action of *S. purpurea* inhibition of HSV-1 replication, the extract was added to a viral single-cycle growth experiment at varying times post-infection. Untreated virus produced an approximate 3.5-log increase in viral titer compared to input virus (Fig. 2). When *S. purpurea* extracts were added at 0 and 0.5 h.p.i., no detectable virus was present after the 24-h growth period. When the extract was added at 1, 4 or 6 h.p.i., an approximate 4–5-log reduction in viral titers was observed (Fig. 2). Notably, the titers of HSV-1 when treated at 1, 4, and 6 h.p.i. were similar to or below the initial input virus titer. The difference in output viral titers between treatment at 0 and 0.5 h.p.i. compared to treatment at 1, 4, and 6 h.p.i. may suggest the *S. purpurea* extract can inhibit HSV-1 replication at two distinct steps in the viral replication process.

### *S. purpurea* extract inhibited HSV-1 attachment to host cells

Since *S. purpurea* extracts inhibited HSV-1 replication when added at the time of infection and the reduction in viral titers were below that of input virus (Fig. 2), it may suggest that the extract blocks viral attachment to the host cell receptor.

**Figure 3:**
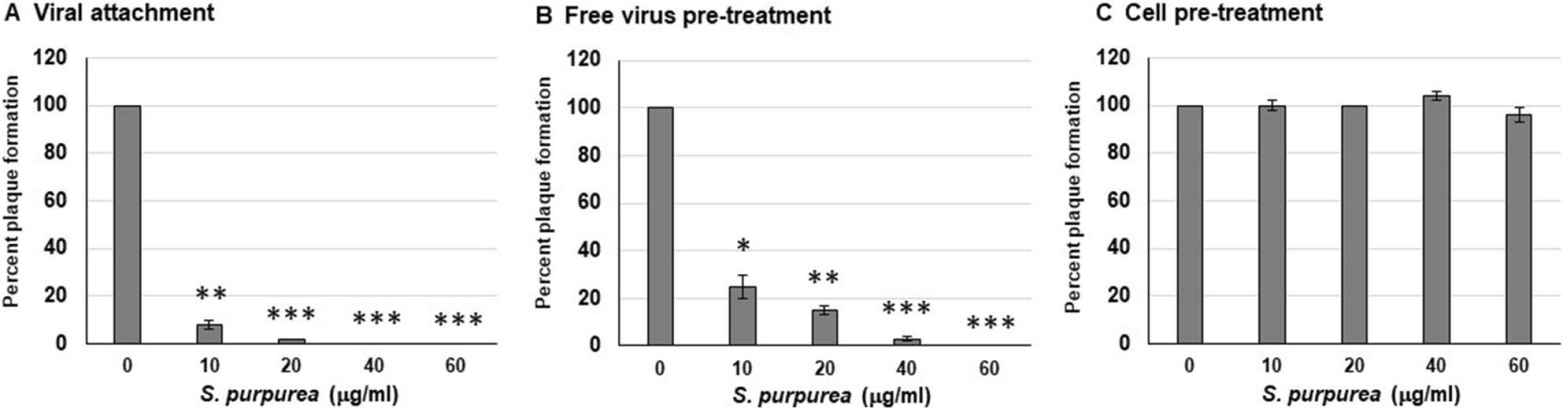
*S. purpurea* inhibited HSV-1 attachment to host cells. (**A**) For the viral attachment assay, Vero cells were infected with 200 pfu HSV-1 in the presence of 0, 10, 20, 40, or 60 µg/ml *S. purpurea* extract and incubated on ice for 2 h. The cell monolayers were washed three times with cold media, followed by the addition of warm media and incubation for 3 days. Plaque formation was visualized by staining with crystal violet. (**B**) For free virus pre-treatment, 200 pfu of purified HSV-1 virions were treated with 0, 10, 20, 40, or 60 µg/ml *S. purpurea* extract and incubated at room temperature for 1 h. After incubation the samples were centrifuged at 20,000 × *g* for 1 h to pellet the virus. The pelleted virus was washed and titered by a standard plaque formation assay. (**C**) For cell pre-treatment, Vero cells were treated with 0, 10, 20, 40, or 60 µg/ml of *S. purpurea* extract and incubated for one hour at 37 °C. The monolayers were washed three times to remove the *S. purpurea* extract. The *S. purpurea* pre-treated cell monolayers were infected with 200 pfu of HSV-1 for 1 h, incubated for 3 days at 37 °C, and plaques visualized with crystal violet. Statistical analysis was performed using a paired t-test. Error bars indicate the standard deviation from three separate trials. Samples with statistically significant deviation relative to the 0 μg/ml *S. purpurea* treatment are indicated with asterisks (*p < 0.05, **p < 0.01, ***p < 0.005).

To examine this further, a viral-cell attachment assay was performed. Vero cells were infected and treated with increasing concentrations of *S. purpurea* extract and incubated on ice for 2 h. Incubation at 4 °C allows for viral binding to the host cell receptor but inhibits viral uptake into the cell. After incubation, the unbound virus and extract was washed away and plaquing level determined. As shown in Fig. 3A, incubation of HSV-1 with *S. purpurea* extract during the viral-cell attachment phase inhibited viral plaque formation suggesting that the extract inhibited viral binding to the host cell. To examine this further, free HSV-1 virions were incubated with the extract, followed by washing of the virus and subsequent infection. As shown in Fig. 3B, the extract was able to inhibit HSV-1 plaque formation when the extract was only incubated with the free virus. Similarly, when Vero cells were pre-treated with the *S. purpurea* extract, washed and then infected with HSV-1, no reduction in viral replication was observed (Fig. 3C). All the experiments performed in Fig. 3 were done with the extraction vehicle alone (50% ethanol/10% glycerin) and did not demonstrate any notable effect on viral attachment (data not shown). These results may suggest that constituents in the *S. purpurea* extract are potentially binding to the HSV-1 surface glycoprotein(s) and inhibiting viral attachment to the host cell or disrupting the virion envelope/structural integrity.

### *S. purpurea* extract inhibited HSV-1 infection at the RNA transcription level

**Figure 4:**
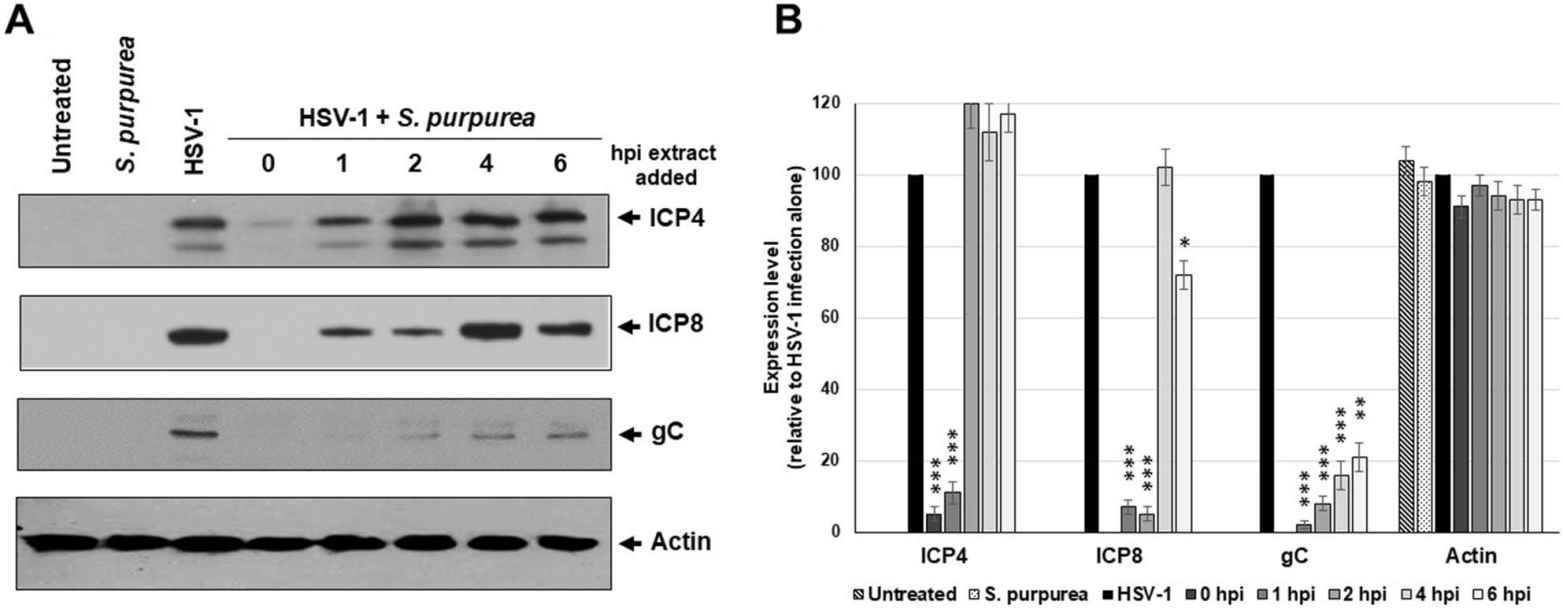
*S. purpurea* reduced HSV-1 ICP4, ICP8, and gC protein levels in a time dependent manner. Vero cells were mock infected or infected with HSV-1 at a MOI = 5 in the presence or absence of *S. purpurea* (40 µg/ml) added at 0, 1, 2, 4, and 6 h.p.i. Cells were harvested at 16 h.p.i., lysed, separated by SDS-PAGE analyzed by Western blot with antibodies to HSV-1 ICP4, ICP8, gC and cellular actin. Actin was included as a standard loading control. (**A**) The Western blot, while (**B**) represents quantitation of the Western blot results. Error bars indicate the standard deviation from three separate analyses. Statistical analysis was performed using a paired t-test. Samples with statistically significant deviation relative to the untreated HSV-1 sample are indicated with asterisks (*p < 0.05, **p < 0.01, ***p < 0.005). Images of the full-length Western blots are included in the “Supplemental files”.

The results from Fig. 2 suggest that the *S. purpurea* extract can not only inhibit HSV-1 attachment to the host cell but also inhibit viral replication intracellularly when added after viral uptake into the cell. When Vero cells were treated with *S. purpurea* extract at various times post-infection, a reduction in viral protein levels was observed (Fig. 4A,B). When the extract was added at 0 or 1 h.p.i., a significant reduction in the level of the immediate early protein, ICP4, was observed (Fig. 4A,B). For the early protein, ICP8, addition of the extract even at 2 h.p.i. significantly reduced the level of ICP8 (Fig. 4A,B). For the late protein, gC, treatment with the extract through 6 h.p.i. significantly reduced the level of this protein (Fig. 4A,B). These results agree with the temporal synthesis of these proteins, where depending on the cell line, immediate-early protein synthesis begins by 30 min post-infection, early protein synthesis begins around 2–3 h.p.i and late protein synthesis begins around 6–8 h.p.i.^54,55^. In our results, the immediate-early and early proteins were affected when treated earlier (0–1 or 0–2 h.p.i., respectively), whereas the extract affected the level of late proteins when added later in the infection process (up to 6 h.p.i.). Notably, treatment with the extraction vehicle alone had no effect on viral protein synthesis (data not shown).

**Figure 5:**
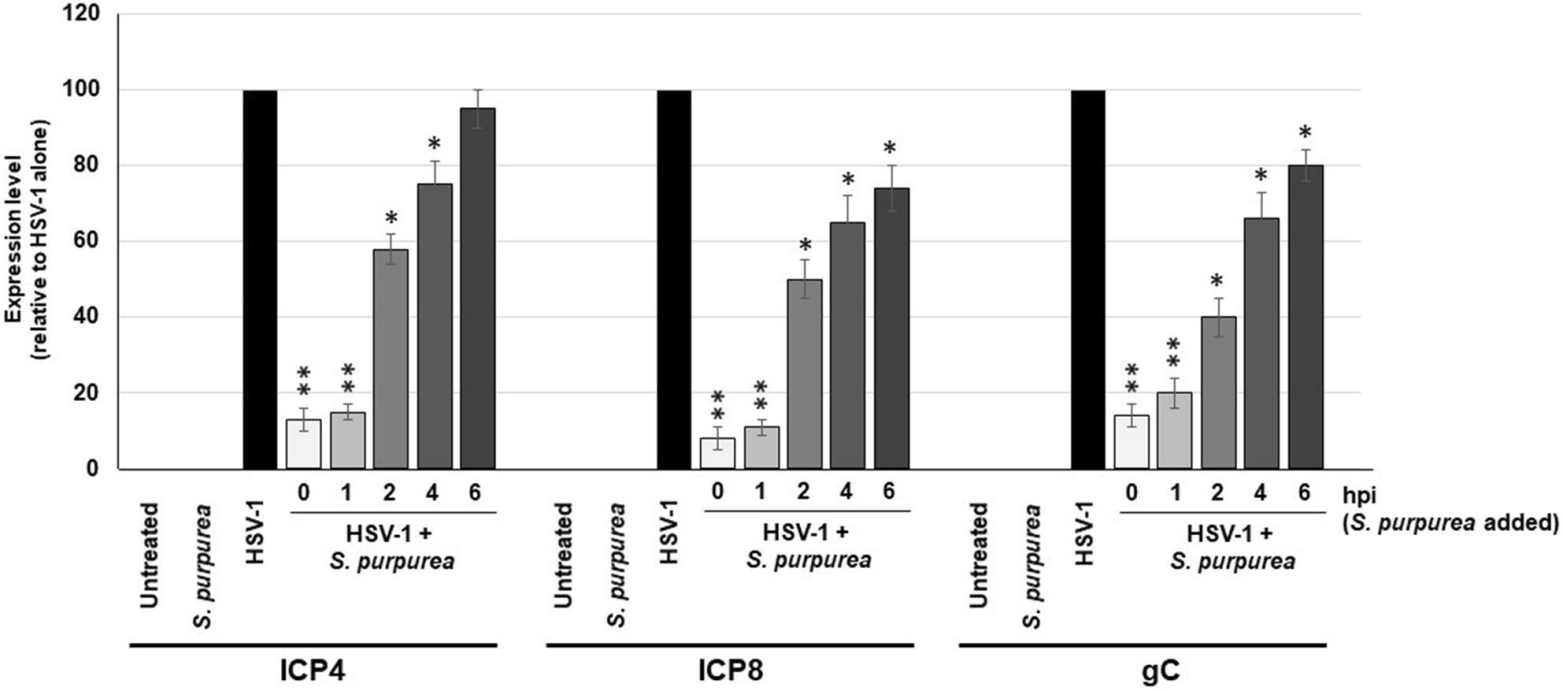
*S. purpurea* inhibited HSV-1 ICP4, ICP8, and gC gene expression. Vero cells were mock infected or infected with HSV-1 at a MOI = 5 in the presence or absence of *S. purpurea* (40 µg/ml) added at 0, 1, 2, 4, and 6 h.p.i. Cells were harvested at 8 h.p.i. and total RNA was isolated. The level of HSV-1 ICP4, ICP8, and gC gene expression was analyzed by real-time PCR. Cellular GAPDH was used as an internal reference and normalization. Error bars indicate the standard deviation from three separate trials. Statistical analysis was performed using a paired t-test. Samples with statistically significant deviation relative to the untreated HSV-1 sample are indicated with asterisks (*p < 0.05, **p < 0.01, ***p < 0.005).

Our lab has previously shown that extracts from *S. purpurea* can inhibit viral transcription of poxviruses^34^. The reduced level of HSV-1 viral proteins following treatment with *S. purpurea* (Fig. 4) could be due to an inhibition of viral transcription. To test for this, Vero cells were infected with HSV-1, treated with *S. purpurea* extracts at 0, 1, 2, 4, and 6 h.p.i., followed by purification of the RNA at 8 h.p.i. RT-PCR was done to determine the levels of HSV-1 ICP4, ICP8, and gC genes and normalized to cellular GAPDH. Results demonstrated that extracts of *S. purpurea* inhibited ICP4 gene expression most effectively following treatment at 0 and 1 h.p.i. (~ 85% reduction) (Fig. 5). ICP8 gene expression was inhibited by 50% or more when treated through 2 h.p.i. and gC gene expression was inhibited by 50% or more through approximately 3 h.p.i. (Fig. 5). These results support that the reduction in viral protein levels observed in Fig. 4 were likely associated with the *S. purpurea* extract inhibiting HSV-1 immediate-early, early and late viral gene expression.

## Discussion

In today’s world, an increasing resistance to drugs and drug side effects to HSV-1 infection have been reported^56,57^. Therefore, finding novel anti-herpes compounds is of critical interest. Current therapeutic drugs, such as acyclovir and its derivatives, have been used in the treatment of HSV-1 infection, however, these drugs are expensive and can result in viral resistance in patients with AIDS and patients with drug-induced immunosuppression. Historically, several plants have been used in traditional-medicine to effective treat HSV-1 infection^22–33^.

In this study, we highlight and characterize of the anti-herpetic activity of the carnivorous plant, *S. purpurea*, which has been reported to relieve pain, lesions and symptoms linked with HSV-1 infection^38–40^. Our lab has previously demonstrated the ability of *S. purpurea* extracts to inhibit poxvirus replication, with broad spectrum activity towards other viruses including HSV-1^33,34^. In this study, we demonstrate that *S. purpurea* extracts inhibited HSV-1-induced CPE, plaque formation and single-cycle growth in a dose-dependent manner. No cell toxicity was observed with *S. purpurea* extracts at the doses used (up to 120 µg/ml) (Fig. 1 and^34^). These results, along with our previous study, support that the *S. purpurea* extract contains bioactive anti-herpes components with limited or no cell toxicity at the doses tested^34^.

For anti-poxvirus activity, *S. purpurea* extracts were previously shown to target and inhibit early viral gene transcription. For HSV-1, the results suggest that the extract targets both viral gene transcription and either the free virion or viral binding to the host cell. Extracts from the botanical, *Melissa officinalis*, have been shown to inhibit HSV-1 binding to cells^32^. These extracts contain an active constituent which binds to the HSV-1 surface gB thereby inhibiting interaction with the host cell receptor. The active constituent(s) in *M. officinalis* is caffeic acid and/or its derivatives^58^. Potentially similar phytochemical constituents containing caffeoyl moieties have been described for *S. purpurea*^59^. Though speculative, the caffeoyl moiety containing constituents present in *S. purpurea* extracts may act similarly to the compounds present in *M. officinalis* by binding to the HSV-1 surface glycoproteins. Alternatively, constituents present in the *S. purpurea* extract may interact with the free virion directly and disrupt the integrity of the envelope. This has been observed previously with other botanical constituents, including curcumin, which has been shown to disrupt the integrity of the viral envelope of several viruses^60^. Since previous work using *S. purpurea* extracts against poxviruses demonstrated that the extract did not disrupt the poxvirus envelope^34^, we propose that the extract is likely blocking HSV-1 attachment to the cell, although further studies to confirm this are required.

The results presented also support that the *S. purpurea* extract inhibited replication of HSV-1 at a point following viral uptake into the host cell. When the extract was added to viral infected cells up to 6 h.p.i, viral replication was inhibited (Fig. 2). As mentioned, *S. purpurea* has previously been shown to inhibit poxvirus replication by inhibiting early viral gene transcription^34^. Treatment with the extract at various stages during HSV-1 replication cycle resulted in a reduction in viral gene expression and a corresponding reduction in viral protein levels. Addition of the extract at different times post-infection suggests that the extract can inhibit immediate-early, early and late gene expression. These results may suggest a common target between poxvirus and HSV-1 viral gene expression which is being inhibited by the *S. purpurea* extract.

In conclusion, the *S. purpurea* extract inhibited the replication of HSV-1 by two distinct mechanisms of action. *S. purpurea* directly inhibited the free virion or viral attachment to host cells, as well as inhibiting the expression of viral gene transcription. These results support a broader anti-viral activity of *S. purpurea* extracts against both pox and herpes viruses. Further research to isolate and identify the distinct constituents leading to these antiviral activities is necessary to confirm these results and further elucidate the mechanism of action. Limited studies support the therapeutic value of *S. purpurea* in treating HSV-1 associated herpes labialis through topical application^38–40^. Isolation of the active constituents present in *S. purpurea* may provide future pharmaceutical therapies for HSV-1, and potentially other, herpes virus outbreaks.

## Supplementary information

Supplementary Figure.
