# Supplementary material for: Anti-herpes virus activity of the carnivorous botanical, Sarracenia purpurea
Source: Sci Rep. 2020 Nov 3;10:18953. doi: 10.1038/s41598-020-76151-w (PMC7609557; doi:10.1038/s41598-020-76151-w)

**Anti-herpes virus activity of the carnivorous botanical, *Sarracenia purpurea***

**Authors**: Latha Kannan, Ashok Kumar, Aradhana Kumar, Bertram Jacobs and Jeffrey Langland

**Supplementary figure. *S. purpurea* reduced HSV-1 ICP4, ICP8, and gC protein levels in a time dependent manner.** Figure shows the full-length Western blots of those shown in Figure 4 of the manuscript. Vero cells were mock infected or infected with HSV-1 at a MOI=5 in the presence or absence of *S. purpurea* (40 µg/ml) added at 0, 1, 2, 4, and 6 h.p.i. Cells were harvested at 16 h.p.i., lysed, separated by SDS-PAGE analyzed by Western blot with antibodies to HSV-1 ICP4, ICP8, gC and cellular actin. Actin was included as a standard loading control.


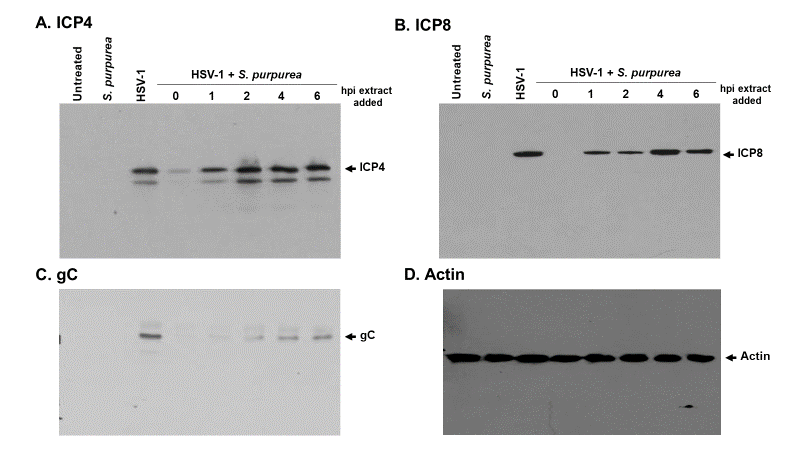

Supplement: Supplementary file 1 — Supplementary Figure. [file 41598_2020_76151_MOESM1_ESM.docx]
